# Mitochondrial Antiviral Signaling (MAVS) Protein Modulates the Transition from Acute to Persistent Parainfluenza Virus Infection and Resistance to Complement-Mediated Cell Lysis

**DOI:** 10.3390/v18040416

**Published:** 2026-03-27

**Authors:** Jenna R. Aquino, Griffith D. Parks

**Affiliations:** Burnett School of Biomedical Sciences, College of Medicine, University of Central Florida, Orlando, FL 32827, USA; jenna.aquino@ucf.edu

**Keywords:** complement, parainfluenza virus, persistent infection

## Abstract

Parainfluenza virus 5 (PIV5) can establish persistent infections in host cells despite encountering innate immune defenses, including the complement (C′) system. The host determinants that enable persistently infected cells (PI) to evade C’-mediated clearance remain largely undefined. Here, we identify the mitochondrial antiviral signaling (MAVS) protein, a central adaptor in double-stranded RNA-triggered antiviral and pro-survival signaling pathways, as a critical mediator of both PIV5 persistence and acquired resistance to C’ lysis. Wild-type (WT) PIV5-infected A549 cells were initially sensitive to C’-directed killing, but these cells rapidly establish a PI in culture with ~25% of the cell population becoming resistant to C’ lysis by day 2 and ~75% by day 4. In contrast, PIV5-infected A549 MAVS-deficient (MAVS KO) cells exhibited elevated viral gene expression, increased deposition of C3 and the membrane attack complex, and were more susceptible than WT cells to C′ killing. PIV5-infected MAVS KO cells showed rapid cytopathic effects and never established a stable PI. While pharmacological suppression of viral gene expression with ribavirin (RBV) restored the survival of PIV5-infected MAVS KO cells into a long-term PI-like state, these RBV-induced PI cells remained sensitive to C’ lysis. Collectively, these findings demonstrate a role of MAVS in modulating a PIV5 infection in culture, to facilitate both the conversion of a PIV5 acute infection to a PI and development of resistance to C’ killing.

## 1. Introduction

Infections with RNA viruses can, in some cases, undergo changes that convert an acute infection (AI) to a persistent infection (PI) where cells remain viable and grow while continuously producing viral antigens and progeny infectious virions [1]. Some of these PIs include important human pathogens such as the hepatitis C virus, measles virus, and SARS-CoV-2 [2,3,4,5,6,7,8,9]. Prolonged and PIs with RNA viruses can present major clinical challenges, including extended damaging inflammation, increased opportunity for spread of infection through human populations, and generation of viral variants that escape immune control. Building an understanding of factors that dictate the AI to a PI progression and how this affects immune recognition is key to developing new approaches to combat these prevalent infections.

The complement (C’) cascade is a major innate immune pathway which can limit virus replication, lyse infected cells, and recruit lymphocytes to the site of infection to promote adaptive immunity [9]. C’ recognizes virus-infected cells as abnormal, resulting in the deposition of C3 fragments followed by the triggering of proteolytic cascades involving C3 and C5 convertases, and ultimately targeting these cells for lysis through the membrane attack complex (MAC) [10,11]. However, many RNA viruses have evolved effective strategies to evade C’ killing. This evasion of immune mechanisms is particularly challenging in the case of viral PIs where front-line immune pathways such as C’ could be inhibited [12,13,14,15,16,17,18,19,20,21].

In previous work, we showed that tissue-cultured cells with an AI by parainfluenza virus 5 (PIV5) can convert from a C’-sensitive AI to a PI cell population characterized by nearly complete resistance to C’-directed lysis [12]. Additionally, PIV5 PI cells exhibit lower levels of viral gene expression and of cell surface glycoproteins compared to AI cells [22]. Pharmacologically reducing viral surface glycoproteins in AI cells to levels seen in PI cells can convert the C’-sensitive AI phenotype to the C’-resistant PI phenotype [22]. These findings suggest that the amount of viral surface glycoproteins present on infected cells at least in part influence their susceptibility to C’ killing. Alternative factors that drive the transition from an AI to a PI and changes in C’-sensitivity remain incompletely understood.

The mitochondrial antiviral signaling (MAVS) protein is a central adaptor in cytosolic RNA-sensing pathways, being activated by RIG-I-like receptors (MDA-5 and RIG-I) which recognize viral double-stranded RNA (dsRNA) [23]. Upon activation, MAVS initiates signaling cascades that lead to IRF-3 phosphorylation and expression of antiviral genes, production of type I interferons (IFN), and synthesis of STAT1-dependent interferon-stimulated genes (ISGs)—thereby restricting viral replication and spread [24]. However, accumulating evidence indicates that MAVS can also promote cellular survival under conditions of sustained viral RNA accumulation. In the case of some paramyxovirus infections, prolonged MAVS activation has been shown to induce a pro-survival transcriptional profile, allowing infected cells to evade cell death while maintaining a PI [25]. Additionally, MAVS has more recently been recognized as a critical regulator of apoptosis, NLRP3 inflammasome activation, and autophagy. [26]. Together, these examples suggest that MAVS can shape the cellular environment during infection through transcriptional modulation, cytokine production, and alteration of viral dynamics, all of which may directly affect whether these AI cells progress to a PI [27].

Given the potential for MAVS to play these dual roles during RNA virus infection, we tested whether genetic ablation of MAVS affects the (i) survival of PIV5-infected cells, (ii) progression to a PI state, and (iii) sensitivity of AI and PI cells to C’-dependent lysis. Using A549 lung cells as a well-developed model system, our results here show that PIV5-infected WT cells efficiently establish a PI with ~25% of the cell population showing resistance to C’ lysis by 2 dpi and ~75% by 4 dpi. By contrast, MAVS knock-out (KO) cells were more sensitive than WT cells to C’-dependent lysis during an AI, and these infected KO cells never progressed to a stable PI phenotype. Together, these findings indicate that MAVS is essential both for facilitating the transition of a PIV5 AI to a PI cell population and for regulating susceptibility of PIV5-infected cells to C’-directed lysis.

## 2. Materials and Methods

### 2.1. Cell Lines, Viruses, and Infections

CV1, Vero, and A549 cells were grown in Dulbecco-modified Eagle medium (DMEM, Gibco, Thermo Fisher Scientific, Waltham, MA, USA) supplemented with 10% heat-inactivated fetal bovine serum (HI FBS, Gibco, Thermo Fisher Scientific). A549 KO-MAVS cells were purchased commercially (A549-Dual™ KO-MAVS cells, catalog #a549d-komavs; InvivoGen, San Diego, CA, USA) and grown in DMEM 10% HI FBS, 100 U/mL penicillin, 100 µg/mL streptomycin, 100 µg/mL Normocin. All cells were maintained at 37 °C under a humidified 5% CO_2_ atmosphere. A549 cells expressing a nuclear red fluorescence protein (A549-NLR, MAVS KO A549-NLR cells) were generated by transduction using NucLight Red lentivirus (Sartorius, Göttingen, Germany) followed by 1 µg/mL puromycin selection. 

The PIV5 Leader mutant (Le-PIV5) encodes two nucleotide substitutions in the genomic promoter (U5C, A14G) and expresses enhanced Green Fluorescent Protein (GFP) from a gene inserted between the HN and L genes, as previously described [28]. The virus was grown in Vero cells and titered on CV-1 cells. Cells were infected at a multiplicity of infection (MOI) of 10, as described previously [4], and cultured in DMEM supplemented with 2% HI FBS.

### 2.2. Quantitative-PCR

Six-well plates of cells were treated as indicated in the figure legends, and RNA extraction was performed using TRIzol (Thermo Fisher Scientific) as described previously [29]. TaqMan^®^ Reverse Transcription Reagents (Applied Biosystems, Foster City, CA, USA) were used to obtain cDNA from 1 µg of total RNA as per the manufacturer’s instructions. Bio-Rad CFX Connect Real-Time and Fast SYBR^®^ FAST Green Master Mix (Applied Biosystems) were used to perform quantitative real-time PCR. The primer sequences utilized are shown in Table 1, below:

### 2.3. Western Blotting

Six-well plates of cells were treated as described in the figure legends and were lysed using SDS and reducing protein lysis buffer. Cell lysates were resolved on 12% sodium dodecyl sulfate-polyacrylamide gel electrophoresis (SDS-PAGE) gels and transferred to nitrocellulose membranes (Bio-Rad, Hercules, CA, USA). After normalizing the samples with Western blotting for β-Actin (1:10,000 dilution, catalog #A5316; Sigma-Aldrich, St. Louis, MO, USA), the samples were then probed with antibodies for STAT1 (STAT1 antibody, 1:1000 dilution, catalog #14994S; Cell Signaling Technology, Danvers, MA, USA). The blots were visualized using anti-mouse horseradish peroxidase (HRP) conjugated antibodies (Sigma-Aldrich) and chemiluminescence (Thermo Fisher Scientific).

### 2.4. Flow Cytometry

Cells cultured in 24-well plates (Corning, Corning, NY, USA) were mock-infected or infected with Le-PIV5 for the specified durations. Adherent cells were trypsinized, combined with media, centrifuged, fixed, and permeabilized using Invitrogen eBioscience (Thermo Fisher Scientific) reagents according to manufacturer’s recommendations. Samples were then stained with propidium iodide (PropI) (BD Bioscience, Milford, MA, USA) as described by the manufacturer. Alternatively, cells were stained with a mouse anti-PIV5 polyclonal antibody (1:500 dilution, [30]), followed by secondary staining with anti-mouse AlexaFluor 405 (1:1000, catalog # A31553; Thermo Fisher Scientific, Waltham, MA, USA).

To measure the deposition of C’ factors, cells were mock-infected or infected with Le-PIV5. At 24 h post infection (hpi), cells were treated with dilutions of normal human serum (NHS; Innovative Research, Novi, MI, USA). Control sera were heat-inactivated (HI) by heating to 56 °C for 30 min. Reactions were incubated for 30 min at 37 °C, followed by a media wash and centrifugation. Cells were then surface-stained with an antibody against MAC (C5b-9) (1:500, catalog # A239; Quidel, San Diego, CA, USA) or C3 (1:1000, catalog # 204869; Calbiochem, San Diego, CA, USA). Primary antibody incubation was followed by secondary anti-mouse AlexaFluor 405 (1:1000, catalog # A31553; Thermo Fisher Scientific, Waltham, MA, USA) or anti-goat AlexaFluor 405 (1:1000, catalog #: A48259; Thermo Fisher Scientific, Waltham, MA, USA). All cell experiments were quantified by flow cytometry using CytoFLEX (Beckman Coulter, Brea, CA, USA) and 10,000 independent events were analyzed using CytExpert software (Beckman Coulter, version 2.4).

### 2.5. Cytotoxicity and Cell Killing Assays

Real-time cell viability assays were performed using an IncuCyte instrument (Sartorious, Göttingen, Germany) as previously described [31]. Briefly, target NLR cells were plated in triplicate in 96-well plates (Corning, Corning, NY, USA) at 7000 cells/well and incubated overnight. Cells were mock-infected or infected with Le-PIV5 for the indicated durations and treated with NHS or HI NHS as a control at 18 hpi. Plates were incubated at 37 °C within the IncuCyte system and images were recorded every 2 h using a 10× objective with red, green, and phase channels. The red object count (ROC) corresponding to cell nuclei was calculated for each well. The values for the ROC at each timepoint were expressed as a percentage of the value at time zero (ROCt^0^) and expressed as ROC/ROCt^0^. Percent cytotoxicity was determined by normalizing the ROC in wells incubated with NHS and target cells to the ROC of control wells containing target cells and HI NHS controls, thereby accounting for differences in cell growth.

### 2.6. Ribavirin Treatments

Cells were treated with ribavirin (RBV, 1-β-d-ribofuranosyl-1,2,4-triazole-3-carboxamide, 196066, MP Biomedicals, Eschwege, Germany) at a final concentration of 10 μg/mL, as described previously [22]. WT and MAVS KO A549 cells were treated with RBV for 24 h prior to Le-PIV5 infection and maintained in RBV-containing media throughout the infection time course.

### 2.7. Statistics

All statistical analyses were performed using Prism GraphPad. Groupwise comparisons were assessed using Student’s *t*-test (2 groups) or analysis of variance (ANOVA; more than 2 groups). Values in all panels are the means of three replicates, with error bars representing standard deviation. In all figures, * indicates a *p* value < 0.05, ** indicates a *p* value < 0.01, *** indicates a *p* value < 0.001, and **** indicates a *p* value < 0.0001.

## 3. Results

### 3.1. Loss of MAVS Impairs Antiviral Gene Induction Independent of STAT1 Degradation

**Figure 1:**
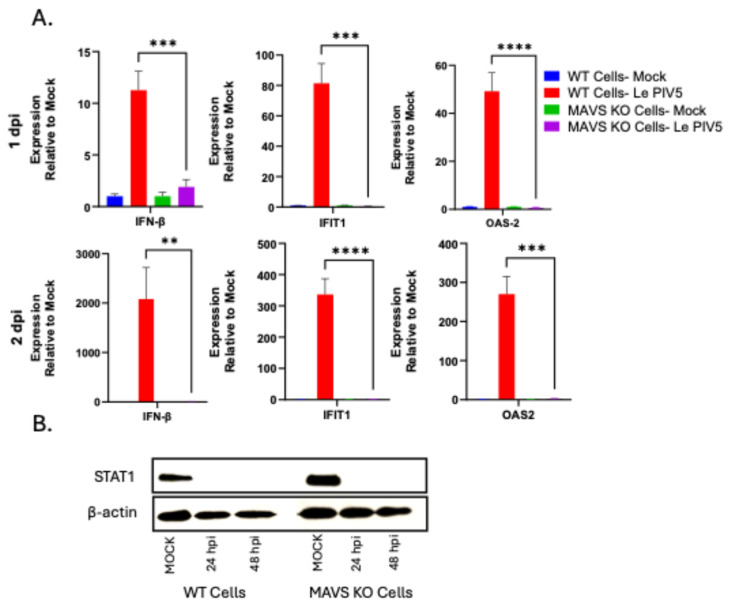
Loss of MAVS impairs antiviral gene induction independent of STAT1. (**A**) WT or MAVS KO A549 cells were mock-infected or infected with Le-PIV5 at an MOI of 10 and harvested at 24 or 48 hpi. Total RNA was analyzed by quantitative PCR to assess expression of IFN-β, IFIT1, and OAS2. Gene expression levels are expressed relative to the corresponding mock-infected controls. (**B**) Protein lysates from WT or MAVS KO cells that were mock-infected or infected with Le-PIV5 at a MOI of 10 were collected at 24 and 48 hpi and analyzed by Western blotting for STAT1 protein. β-actin was used as a loading control. ** indicates a *p* value < 0.01, *** indicates a *p* value < 0.001, and **** indicates a *p* value < 0.0001.

In this study, we utilized the Le-PIV5 mutant due to an enhanced C’ response relative to a wild-type virus and the capacity to activate antiviral signaling despite encoding an intact V protein [11,32]. To validate that MAVS KO cells have an impairment in expression of antiviral genes after Le-PIV5 infection, we performed qPCR on WT and MAVS KO cells that were mock-infected or infected with Le-PIV5. The assay measured expression of key antiviral genes known to be induced by Le-PIV5—including IFN-β, IFIT1, and OAS2. As shown in Figure 1A, mock-infected WT cells showed minimal basal expression of these three antiviral genes (blue bars), which contrasted with high induction of these genes (~12–80 fold) in WT cells infected with Le-PIV5 (red bars). Basal expression in mock-infected MAVS KO cells was comparable to WT controls (green bars); however, MAVS KO cells infected with Le-PIV5 failed to upregulate any of the antiviral genes examined (purple bars). These differences in antiviral gene expression persisted through 48 hpi (Figure 1A).

Given that antiviral genes can be induced through a STAT1-dependent pathway mediated by secreted IFN and that the PIV5 V protein directs STAT1 degradation [33], we tested the hypothesis that observed differences in antiviral gene expression might be due to altered STAT1 levels between WT and MAVS KO cells. Western blot analysis at 24 and 48 hpi demonstrated that STAT1 was present in both mock-infected WT and MAVS KO cells, whereas STAT1 was efficiently degraded in both WT and MAVS KO cells infected with Le-PIV5 at both time points (Figure 1B). Both WT and MAVS KO cells undergo STAT1 degradation upon Le PIV5 infection, indicating that the failure of MAVS-KO cells to induce antiviral gene expression is not due to differences in IFN signaling through STAT1, but rather reflects a defect in MAVS-dependent gene expression.

### 3.2. MAVS-Deficient Cells Show Increased PIV5 Viral Gene Expression and Accelerated Cytopathic Effect

**Figure 2:**
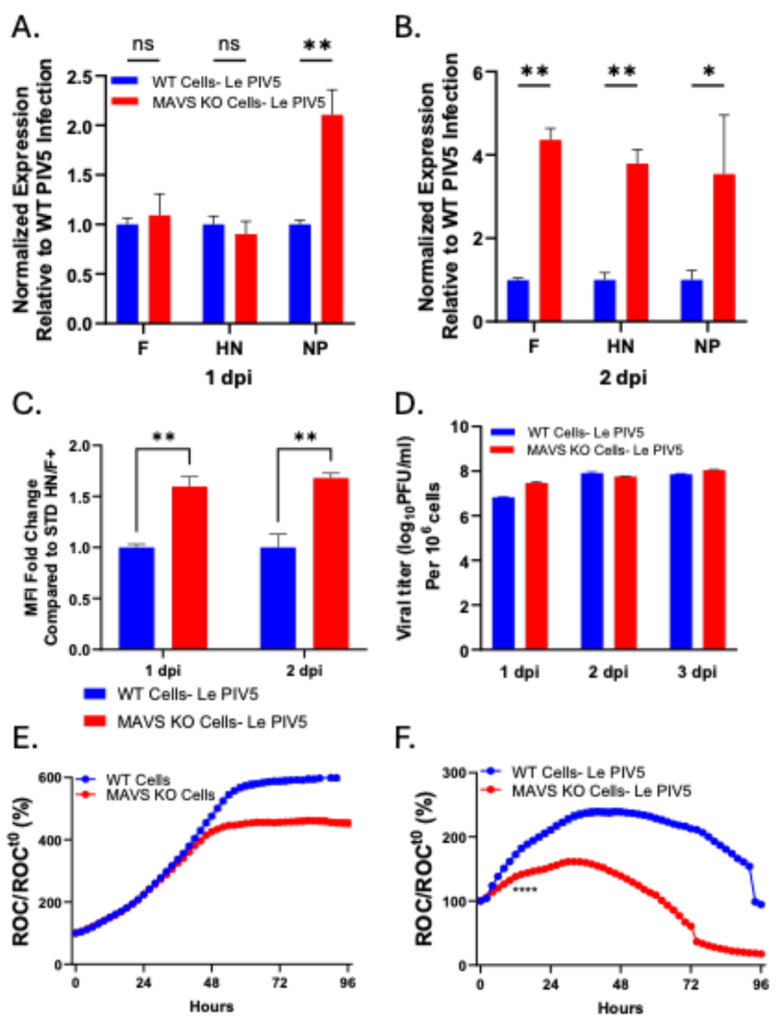
Increased PIV5 viral gene expression and accelerated cytopathic effect in MAVS-deficient cells. (**A**–**D**) WT or MAVS KO cells were mock-infected or infected with Le PIV5 infected at an MOI of 10. (**A**,**B**) Levels of F, HN and NP gene expression were quantified by qPCR at 1 and 2 dpi and plotted relative to Le PIV5–infected WT cells. (**C**) Cell surface levels of PIV5 viral glycoprotein were assessed by staining with polyclonal anti-PIV serum followed by flow cytometry at 1 and 2 dpi of WT or MAVS KO cells. Data are presented as mean fluorescence intensity (MFI) fold change relative to WT-infected cells. (**D**) Cumulative Le-PIV5 viral titers determined by plaque assay at 1, 2, and 3 dpi for WT and MAV KO cells. (**E**,**F**) Cell viability was determined up to 96 hpi for WT and MAVS KO cells that were mock-infected (**E**) or infected with Le-PIV5 (**F**). Red object count (ROC) per well was quantified using the IncuCyte instrument and normalized to the ROC at time zero (ROC/ROCt^0^) when Le-PIV5 infection began, expressed as a percentage of time zero. **** indicates when a *p* value < 0.0001 first appeared on the time course, comparing mock or WT Le PIV5-infected cells to mock or MAVS KO Le PIV5-infected cells. This statistical significance was maintained throughout the remainder of the time course. ns: no significance; * indicates a *p* value < 0.05, ** indicates a *p* value < 0.01, and **** indicates a *p* value < 0.0001.

MAVS-dependent antiviral signaling has been shown to restrict viral gene expression [23]. With MAVS KO cells unable to mount this antiviral response, we determined expression of PIV5 F, HN and NP genes at 1 and 2 days post infection (dpi) at an MOI of 10. As shown in Figure 2A,B, MAVS KO cells showed early elevation in NP expression at 1 dpi, followed by significantly higher expression of all 3 viral genes by 2 dpi. Using a polyclonal anti-PIV5 antibody, cell surface expression of PIV5 glycoproteins was higher in MAVS KO cells compared to WT cells (Figure 2C). Interestingly, this higher level of PIV5 gene expression did not yield higher levels of progeny infectious virus, as seen in Figure 2D where virus titers were only slightly higher in MAVS KO cells at 1 dpi.

We used our previously described real-time cell monitoring assay [11,12] to define the kinetics of cell viability following PIV5 infection of WT and MAVS KO A549 lung cells. A549 cells stably expressing a nuclear red fluorescent protein (A549-NLR) were used in this assay, enabling the IncuCyte system to record red fluorescent nuclei at defined intervals in real time. Every 2 h, the number of red-labeled cells (Red Object Counts, ROC) per well was measured and normalized to the ROC at the start of infection (ROCt^0^). Data were expressed as a percentage of ROCt^0^. When comparing cell growth between mock-infected WT (Figure 2E; blue line) and MAVS KO (Figure 2E; red line) cells, both cell types exhibited similar growth kinetics up to 48 post plating. At this point, the ROC for MAVS KO cells begins to plateau. In the case of Le-PIV5 infections (Figure 2F; red line), MAVS KO cells showed an earlier and more substantial plateauing of ROC, followed by a sharp loss of ROC/ROCt^0^ to less than 10% by ~80 hpi. Collectively, these results indicate that MAVS KO cells sustain a higher-than-WT level of viral expression and cell surface glycoprotein expression during Le-PIV5 infection, which likely contributes to their increased loss of viability.

### 3.3. MAVS KO Cells Are More Sensitive than WT Cells to C’-Directed Killing

Given that Le-PIV5-infected MAVS KO cells show increased viral genome expression and glycoprotein surface levels, we hypothesized that this would render them more sensitive to C′ killing. To test this, WT and MAVS KO cells were either mock-infected or infected with Le-PIV5 for 18 h, after which NHS was added as a source of C’. Loss of cell viability was monitored in real time using the IncuCyte system.

**Figure 3:**
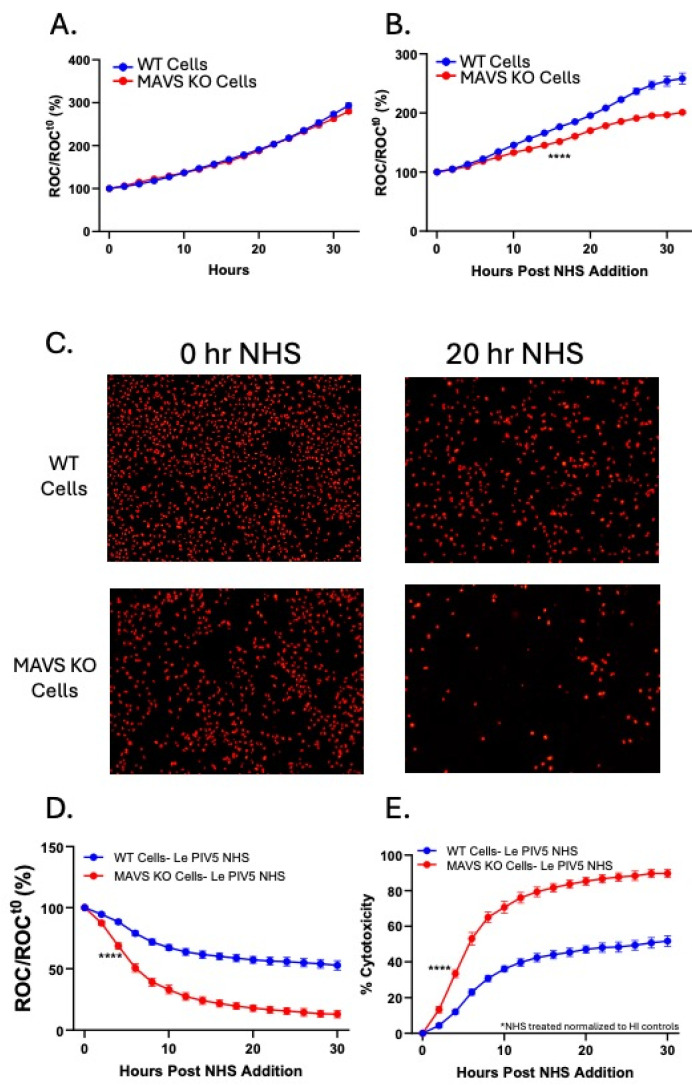
MAVS KO cells are more sensitive to C’ killing compared to WT cells. WT or MAVS KO cells were mock-infected or infected with Le-PIV5 at an MOI of 10, followed by treatment with NHS. The kinetics of cell death were monitored using the IncuCyte system. (**A**) Growth kinetics of mock-infected WT and MAVS KO cells in the absence of NHS. (**B**) Growth kinetics of mock-infected WT and MAVS KO cells in the presence of NHS. (**C**) Representative red fluorescent images of WT or MAVS KO PIV5-infected cells that were treated with NHS were acquired using the IncuCyte at 0 and 20 h following NHS addition. (**D**) Red object count (ROC) per well was quantified over the indicated time period, normalized to the ROC at time zero (ROC/ROC_0_), and expressed as a percentage of the initial value. **** denotes the first time point at which a statistically significant difference (*p* < 0.0001) was observed when comparing WT and MAVS KO mock-infected or PIV5-infected cells treated with NHS; this significance was maintained throughout the remainder of the time course. (**E**) Percent cytotoxicity was calculated using ROC values from NHS-treated wells normalized to the corresponding WT or MAVS KO wells cultured with HI NHS controls. **** indicates a *p* value < 0.0001.

As shown in Figure 3A, in the absence of NHS, both WT and MAVS KO cells displayed similar growth kinetics out to 30 h. In contrast, MAVS KO mock-infected cells exhibited a modest increase in sensitivity to C′ compared to WT cells (Figure 3B), suggesting that MAVS contributes to basal protection of non-infected cells against C’ lysis. Figure 3C shows representative images of Le-PIV5-infected WT and MAVS KO cells at 0 and 20 h after addition of NHS. After addition of NHS, MAVS KO cells infected with Le-PIV5 showed significantly fewer red fluorescent nuclei by 20 h (lower right panel, Figure 3C). Real-time kinetic analysis was carried out on C′ killing, expressed as a percentage of initial cell numbers at the time of NHS addition (ROC/ROCt^0^, %). Le-PIV5-infected MAVS KO cells exhibited a rapid and substantial decrease in ROC upon NHS treatment (Figure 3D; red line), reaching 50% ROC relative to time 0 by 8 h after addition of NHS. This was significantly faster than WT cells (Figure 3D; blue line) which did not reach the same 50% value until >25 h post NHS treatment. When these data were normalized to heat-inactivated (HI) NHS to account for baseline differences in cell growth, the resulting percent cytotoxicity plot (Figure 3E) demonstrated enhanced C′ killing of MAVS KO cells.

**Figure 4:**
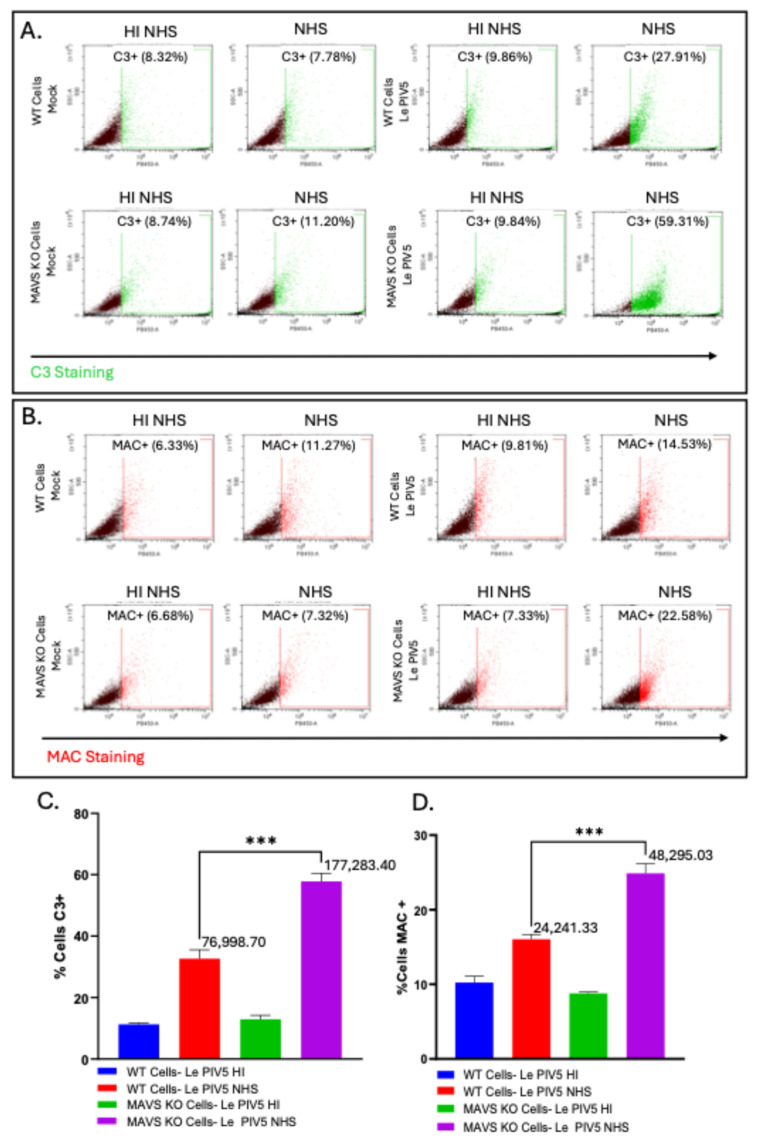
MAVS deficiency leads to increased C3 and MAC deposition during PIV5 infection. WT or MAVS KO cells were mock-infected or infected with PIV5 at a multiplicity of infection (MOI) of 10. At 18 hpi, cells were treated for 30 min with either 20% HI NHS or NHS as a source of C’ factors. (**A**) Following treatment, cells were analyzed by flow cytometry using an anti-C3 antibody to evaluate C3 deposition on WT and MAVS KO PIV5-infected cells. Arrow at bottom of panel indicates increased C3 staining. (**B**) In parallel, cells were analyzed by flow cytometry using an anti-MAC antibody to assess MAC deposition on WT and MAVS KO PIV5-infected cells. Arrow at bottom of panel indicates increased MAC staining. (**C**) Graph represents percentage (%) of cells positive for C3 staining by flow cytometry comparing WT Le PIV5-infected cells to MAVS KO PIV5-infected cells. Corresponding MFI values are present next to Le PIV5-infected WT or MAVS KO cells. (**D**) Graph represents % of cells positive for MAC staining by flow cytometry comparing WT Le PIV5-infected cells to MAVS KO Le PIV5-infected cells. Corresponding MFI values are present next to PIV5-infected WT or MAVS KO cells. *** denotes the first time point at which a statistically significant difference (*p* < 0.0001) was observed when comparing C3 or MAC % positive cells for WT and MAVS KO mock-infected or PIV5-infected cells treated with NHS *** indicates a *p* value < 0.001.

We tested the hypothesis that the increased sensitivity of MAVS KO cells to C’-mediated lysis correlated with enhanced cell surface C3 and MAC deposition. WT and MAVS KO cells were either mock-infected or infected with Le-PIV5. At 24 hpi, cells were treated with NHS for 30 min, followed by flow cytometry using anti-C3 and anti-MAC antibodies. As shown in the representative plots in Figure 4A, C3 deposition was minimal (~8–11%) on the surface of both WT and MAVS KO mock-infected cells. By contrast, Le-PIV5-infected MAVS KO cells showed substantially higher C3 positivity (59%) compared to infected WT cells (28%). Similarly, MAC staining remained minimal in both mock-infected WT and MAVS KO cells (Figure 4B), but there was a substantial difference in the case of Le PIV5-infected MAVS KO cells which displayed an increase in MAC deposition (23%) compared to infected WT cells (15%). Figure 4C shows the percentage of cells positive for C3, revealing a significant increase in C3 deposition on Le-PIV5-infected MAVS KO cells compared with WT cells. Consistent with this observation, C3 MFI was significantly higher for infected MAVS KO cells (177,283) than infected WT cells (76,998). Similarly, Figure 4D shows the percentage of cells positive for the MAC, demonstrating an increase in MAC-positive cells among infected MAVS KO cells compared to WT cells. Infected MAVS KO cells exhibit a higher MAC MFI (48,295) compared with infected WT cells (24,241). Taken together, these results indicate that loss of MAVS not only leads to increased viral gene expression but also amplifies the infected cell’s susceptibility to C3 deposition, MAC deposition, and C’ cytotoxicity.

### 3.4. Differential Capacity of WT and MAVS KO Cells to Establish a Persistent PIV5 Infection

Our previous work demonstrated that a Le-PIV5 AI can transition to a PI, and this correlates with these PI cells gaining resistance to C’ killing [12]. For these A549 PIV5 PI cells, approximately 90% of the cell population was positive for viral gene expression, producing viral proteins and infectious virions at ~3–4-fold and 2.5 logs lower levels than seen in a typical acute infection, respectively. Compared to C’-treated PIV5 AI cells, C3 and MAC deposition on PI cells were decreased by ~50%.

**Figure 5:**
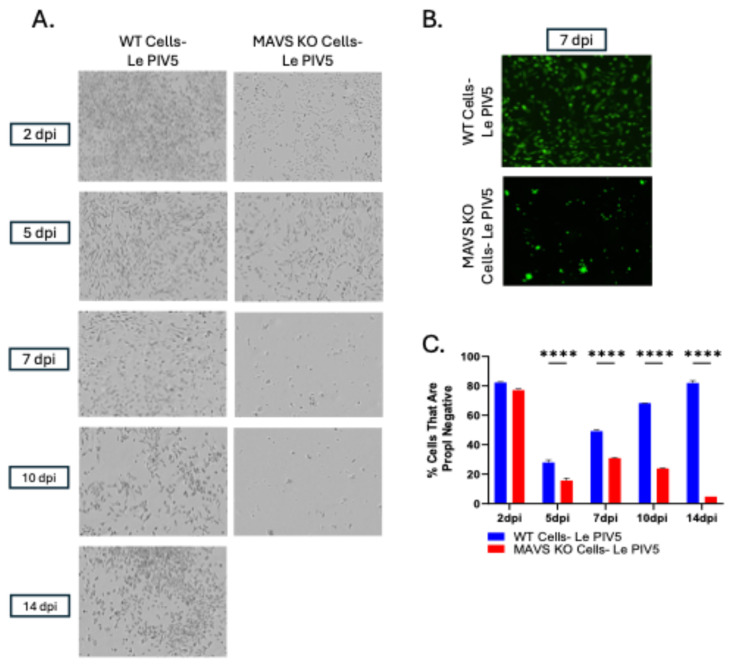
Differential capacity of WT and MAVS KO cells to establish a persistent PIV5 infection. (**A**–**C**) WT or MAVS KO cells were mock-infected or infected with Le-PIV5 at an MOI of 10, and (**A**) bright-field or (**B**) GFP images were acquired at the indicated dpi. (**C**) In parallel, cells were analyzed by flow cytometry for viability using PropI staining. Gating was performed to quantify the number of viable cells that were negative for PropI staining (PI^−^ cells). Data are presented as the percentage of viable (PI^−^) cells remaining at each dpi. **** indicates a *p* value < 0.0001.

To determine if Le-PIV5 could establish a PI in MAVS KO cells, WT and MAVS KO A549 cells were mock- or infected with Le-PIV5 (MOI 10), and cell viability was assessed at 2, 5, 7, 10, and 14 dpi using brightfield imaging and PropI staining. As shown in the representative images of Figure 5A, WT cells initially exhibit cytopathic effects (CPE) up to 5 dpi, including cell rounding and detachment. However, WT cells began to recover by 7 dpi and continued to proliferate and maintain viability by 14 dpi. In contrast, infected MAVS KO cells showed a substantial decline in viable cell numbers out to 5 dpi, with almost no remaining cells by 7 and 10 dpi. Figure 5B shows representative GFP images for both WT and MAVS KO Le-PIV5-infected cells at 7 dpi. Using PropI staining for viable cells with intact plasma membranes, both WT and MAVS KO cells showed a marked reduction in PropI-negative cells by 5 dpi, with only 15–25% of cells remaining viable (Figure 5C). By 7 dpi, over 40% of WT cells were PropI-negative and this continued to increase to 60% by 10 dpi and 80% by 14 dpi. In stark contrast, the number of PropI-negative MAVS KO cells did not expand and remained low or undetectable by 10–14 dpi. These observations indicate that in the absence of MAVS, PIV5-infected A549 cells fail to recover from the AI and are unable to establish a PI.

**Figure 6:**
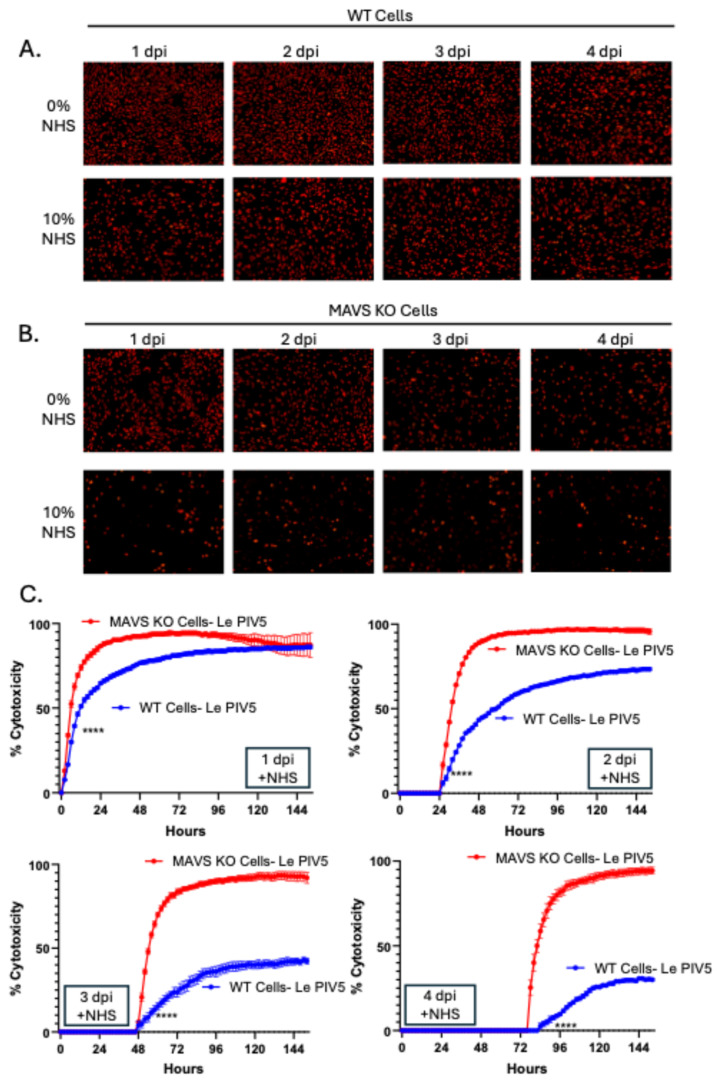
Infected WT cells but not MAVS KO cells gain resistance to C’ killing. WT or MAVS KO cells were mock-infected or infected with Le-PIV5 at a MOI of 10. Separate cultures of infected cells were treated with NHS at 1, 2, 3, or 4 dpi, and cells were monitored by the IncuCyte system. (**A**,**B**) Representative red fluorescent images of PIV5-infected WT or MAVS KO cells that were left untreated or treated with 10% NHS at each indicated time point were acquired, with images collected 24 h after NHS addition. (**C**) ROC was acquired every 2 h after plating of cells in the IncyCyte. These data were calculated by normalizing ROC values from NHS-treated wells to the corresponding WT or MAVS KO wells cultured in the absence of NHS at the same time point of NHS addition. **** denotes the first time point at which a statistically significant difference (*p* < 0.0001) was observed when comparing WT and MAVS KO mock-infected or PIV5-infected cells treated with NHS.

Although MAVS KO cells fail to establish a stable Le-PIV5 PI, we tested whether these cells show any onset of resistance to C’-directed lysis at earlier stages of infection. WT or MAVS KO cells were mock-infected or infected with Le-PIV5 (MOI 10), and NHS was added at a final concentration of 10% at 1, 2, 3, and 4 dpi. Cell killing efficiency was monitored using the IncuCyte. As shown in the representative images in Figure 6A, infected WT cells were initially sensitive to C’-dependent lysis when NHS was added at 24 hpi, consistent with our prior published results [4]. However, over subsequent dpi, the extent of C’-directed lysis of WT cells gradually decreased. In contrast, MAVS KO cells remain strikingly sensitive to C’-directed lysis at every day tested (Figure 6B).

To determine the real-time kinetics and extent of C’-directed lysis, WT or MAVS KO cells were mock-infected or infected with Le-PIV5, incubated in the IncuCyte instrument, and ROC was measured every 2 h. Cytotoxicity was determined through normalization of ROC values from NHS-treated samples to time-matched HI NHS controls. As shown in Figure 6C, treating infected MAVS KO cells with C’ immediately after placing in the IncuCyte (red line) showed more rapid and more extensive C’-directed killing than WT cells (blue line), matching data in Figure 3 above. Most importantly, when cultures were treated with C’ at 24, 48 and 72 h after placing in the instrument, WT cells showed a progressively lower percent cytotoxicity—only ~40% and ~25% cytotoxicity was seen for infected WT cells at 48 and 72 hpi. In sharp contrast, infected MAVS KO cells (red lines) never gained resistance to C’-directed lysis. These data indicate that even before their eventual collapse, MAVS KO cells fail to initiate the early steps toward C’ resistance that are seen in WT cells.

### 3.5. Ribavirin Treatment Allows Le-PIV5-Infected MAVS KO Cells to Survive and Establish a Pseudo-PI State

**Figure 7:**
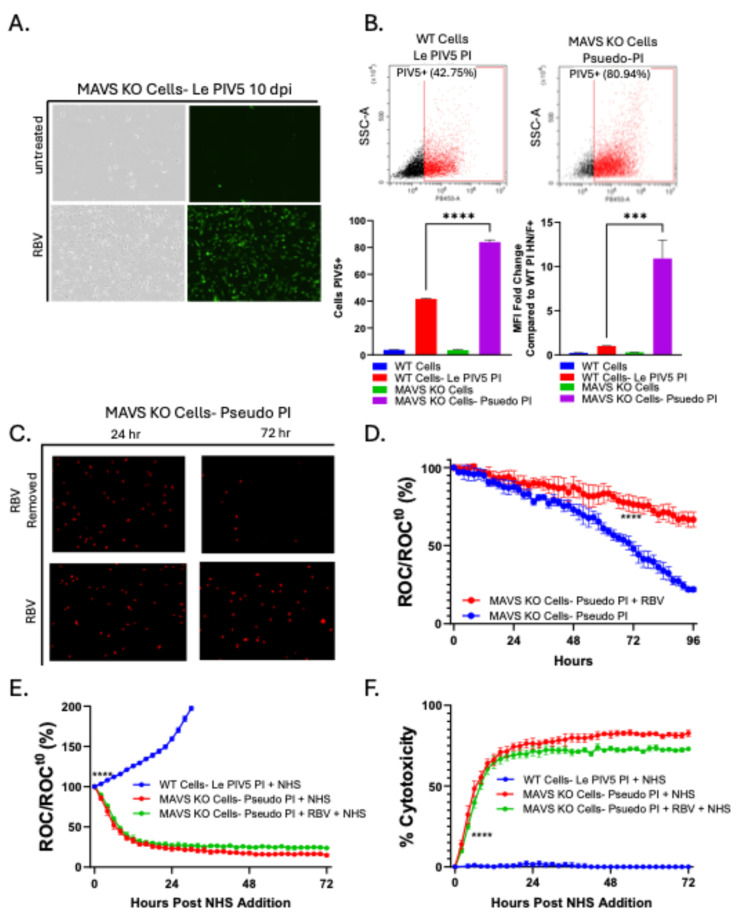
Ribavirin treatment allows Le-PIV5-infected MAVS KO cells to survive to a pseudo-PI state and remain sensitive to C’ lysis. MAVS KO cells were pre-treated with 10 µg/mL of Ribavirin (RBV) prior to Le-PIV5 infection and then cultured with RBV. (**A**) Representative bright-field and GFP images of RBV-treated and untreated MAVS KO cells at 10 dpi. (**B**) Cell surface PIV5 glycoprotein levels were assessed in WT PI cells versus RBV-treated MAVS KO pseudo-PI cells using flow cytometry with an anti-PIV5 antibody. Top panels show representative data as the percentage of cells expressing PIV5 antigens. Bottom panels show mean percent positive cells and mean fluorescence intensity (MFI) of RBV-treated pseudo-PI MAVS KO cells compared to WT PI cells. (**C**) Representative images at 24 and 72 h following maintenance or removal of RBV from MAVS KO pseudo-PI cultures were captured. (**D**) Red object count (ROC) per well was quantified, normalized to the initial ROC (ROC/ROC_0_), and expressed as a percentage of the starting value. Cultures were treated with NHS and analyzed using the IncuCyte instrument. (**E**) ROC per well was quantified for WT PI cells, RBV-treated pseudo-PI MAVS KO cells, and pseudo-PI MAVS KO cells where RBV was removed. Data were normalized to the initial ROC and expressed as a percentage. (**F**) Percent cytotoxicity was generated from the data in panel (**E**), reflecting C’ cell death, shown for each cell type following NHS treatment. ROC values from NHS-treated wells were normalized to the corresponding untreated WT or MAVS KO cells to account for differences in cell growth. **** denotes the first time point at which a statistically significant difference (*p* < 0.0001) was observed between RBV-treated MAVS KO pseudo-PI PIV5-infected cells and cells from which RBV was removed or when comparing WT PI cells to RBV-treated or untreated pseudo-PI MAVS KO cells under NHS treatment. *** indicates a *p* value < 0.001, and **** indicates a *p* value < 0.0001.

Our previous work demonstrated that in acute Le-PIV5-infected WT A549 cells, treatment with Ribavirin (RBV) reduced cell surface viral glycoprotein expression to levels comparable to PI cells, and this converted these C’-sensitive AI cells to C’-resistant cells [22]. Based on this, we hypothesized that suppressing viral gene expression in infected MAVS KO cells with RBV would potentially enable MAVS KO cells to survive and establish a PI culture. MAVS KO cells were cultured with or without 10 µg/mL RBV prior to infection with Le-PIV5, and cell survival was monitored over time. Representative images at 10 dpi (Figure 7A) show that few cells survived in the case of infected and untreated MAVS KO cells, similar to data in Figure 5 above. In contrast, Le-PIV5-infected MAVS KO cells that were cultured with RBV displayed a substantial increase in survival of GFP-positive cells—a pseudo-PI state. Flow cytometry using an anti-PIV5 antibody showed that 80% of RBV-treated MAVS KO pseudo-PI cells were positive for PIV5 antigens (Figure 7B), with an MFI ~10-fold higher than seen with WT PI cells. To test whether continuous RBV treatment was required for survival, RBV was removed from MAVS KO pseudo-PI cultures. As shown in Figure 7C, removal of RBV caused substantial loss of viability, with nearly all cells lost within 72 h. Using real-time IncuCyte assays, when this data is plotted as ROC/ROCt_0_ as in Figure 7D, the divergence in cell growth kinetics between RBV-treated and untreated MAVS KO pseudo-PI cells is further highlighted.

The ability of RBV-treated Le-PIV5 infected MAVS KO cells to survive in a pseudo-PI state raised the question of whether these cells had gained resistance to C’-directed lysis. WT and MAVS KO pseudo-PI cells were treated with 10% NHS, with MAVS KO pseudo-PI cells maintained either with or without RBV and surviving cells were assayed by IncuCyte assays. As expected, WT PI cells resisted C’ lysis after NHS treatment. However, MAVS KO pseudo-PI cells underwent complete C’ lysis regardless of RBV presence. When the data are plotted as the percent of cells remaining relative to time 0 in Figure 7E, WT PI cells (blue line) show robust growth following NHS treatment, whereas MAVS KO pseudo-PI cells exhibit a dramatic loss of viability both in the absence (red line) and presence (green line) of RBV. Similarly, when the data are represented as percent cytotoxicity, the pronounced effect of NHS on MAVS KO Psuedo-PI cells under both conditions is readily apparent (Figure 7F). These results demonstrate that while RBV enables MAVS KO cells to establish a prolonged infection with some properties of a PI, these cells remain fully sensitive to C’ killing. This indicates that MAVS is critical not only for persistence but also for viral evasion of the C’ system in a PI model.

## 4. Discussion

Persistent RNA virus infections represent a major clinical challenge due to prolonged timeframes for spread, continuous inflammatory responses, and emergence of variants which can evade host immune defenses [1,2,3,4]. Clinical and experimental data from persistent RNA viruses demonstrate that prolonged infection can drive chronic pathology, including organ damage, and sustained immune perturbation [5,6,7,8,9,34]. Understanding the host factors that control viral persistence is therefore critical for developing strategies to limit long-term disease. Prior work with cell culture and animal models demonstrates that MAVS ablation leads to uncontrolled viral replication, heightened cytopathic effects, and reduced cell survival [35]. In MAVS-deficient mice, vesicular stomatitis virus (VSV) infection leads to increased disease severity [36]. Given the role of MAVS in cell survival [25] and in the antiviral response to PIV5 infection [32], we tested the hypothesis that MAVS contributes to the establishment of PIV5 C’-resistant PI cells.

Our most striking finding here is that, while cells lacking MAVS had higher-than-WT levels of viral gene expression, unlike WT cells, these infected KO cells fail to transition from an AI to a PI. These results raise the hypothesis that MAVS-dependent antiviral genes that respond to PIV5 infection keep viral RNA synthesis in check and at a less-than-maximal level, thus facilitating the emergence of PI cells in the population. Supporting this idea, previous studies have shown that reduced viral genome expression, through specific phosphorylation sites in the PIV5 P protein, can promote persistence. PIV5 variants with substitutions that prevent phosphorylation at P protein residues 157 and 308 display dampened viral gene expression and enhanced persistence [37]. Furthermore, it has been observed that P protein phosphorylation is not strictly required for the downregulation of viral transcription and replication, as persistent PIV5 cultures can still be established despite variability in P phosphorylation [38]. Together, these observations highlight the critical role of viral gene expression in facilitating the AI to PI transition, whether controlled directly by P protein phosphorylation or indirectly via MAVS-dependent antiviral signaling pathways.

As MAVS is activated through MDA-5 and/or RIG-I sensing of viral dsRNA [23], the level of viral gene expression per se may not be the key factor leading to induction of antiviral genes, but rather the trigger may be levels of aberrant viral RNAs such as dsRNA that arise during viral transcription and genome replication. This would be consistent with our prior results [32] showing that the Le-PIV5 mutant overexpresses viral mRNA and activates RIG-I-dependent antiviral genes, but importantly, these responses are quenched when a dsRNA-binding protein is expressed during the AI.

The increased C3 deposition and C’-mediated lysis in MAVS KO cells may result either from a direct effect of MAVS loss or an indirect consequence of increased viral antigen, consistent with previous observations linking surface antigen levels to C’ sensitivity. To help clarify whether MAVS directly modulates C’, future work should assess MAVS KO cells for C’ regulators (e.g., CD46, CD55, CD59, Factor H), activation markers (C3 cleavage products), and host signaling pathways that control these regulators. Alternatively, it is possible that MAVS normally regulates responses to stress, and this predisposes infected cells to more C’ killing.

It has been previously shown that infection with stocks of the paramyxovirus Sendai virus (SeV) that are enriched in defective interfering (DI) genomes can, as expected, lead to an expression of antiviral genes through MAVS-dependent pathways [25]. Unexpectedly, however, these DI-enriched SeV infections also promote MAVS-dependent expression of pro-survival cellular genes such as TNFR2, TRAF1, and TNFAIP3 [25]. Thus, an alternative model to explain the lack of transition of Le-PIV5-infected MAVS-KO cells from an AI to PI is that key MAVS-dependent pro-survival genes are not expressed to promote progression to stable cell viability. These two models of MAVS-dependent quenching of viral RNA expression and induction of pro-survival genes are not mutually exclusive, and it may be important that, in order for PIV5-infected cells to progress to PI status, they need a balance of suppressed viral RNA synthesis in the context of cells with elevated pro-survival genes. Importantly, PIV5 and SeV encode different viral antagonists of antiviral responses and target different host cell responses [39]. It will be of interest to determine if these two negative-strand RNA viruses have similar or divergent dependence on MAVS for establishing a PI.

We have operationally defined a PIV5 PI in vitro as infected viable cells which can be successfully cultured through multiple passages and which still express viral genes and produce progeny virions [12]. The time course shown here in Figure 5 demonstrates that a Le-PIV5 AI culture of WT cells undergoes a decrease in viable cell number out to 5 dpi, but subsequently, these infected viable cell numbers rebound to AI levels by 12–14 dpi. Time-of-addition experiments with NHS (seen in Figure 6) show that ~25% of Le-PIV5 WT infected cells acquire resistance to C’ lysis as early as 2 dpi and that ~75% of cells are resistant to C’ killing by 4 dpi. By contrast, Le-PIV5-infected MAVS KO cell numbers continued to decrease below AI levels and were always found to be fully sensitive to C’-driven lysis. Importantly, these data are consistent with our prior proposal that acquiring resistance to C’-mediated cell lysis could represent a biomarker for established PIV5 PI [12]. It remains to be determined if C’-resistance as a PI biomarker also applies to other RNA virus infections.

Pharmacologic suppression of viral gene expression with RBV enabled MAVS KO cells to survive long enough to enter a pseudo-PI state—a state where cells remain viable, infected, and can be passed in culture. Removal of RBV caused rapid loss of viability for most of the pseudo-PI MAVS KO cells. These RBV-induced MAVS KO pseudo-PI cells remained highly sensitive to C’-driven cell lysis—in stark contrast to WT cells harboring a PI. These observations indicate that, while decreased viral gene expression is a hallmark of PIV5 PIs, it is not strictly sufficient for entry into a PI state. It appears that other processes driven at least in part by MAVS functions are essential for remodeling the host cell into a C’-resistant, functionally stable PI.

Our results with complete ablation of MAVS in KO cells raise the clinically important question of whether natural human genetic variation in MAVS similarly influences establishment of viral persistence and C’ resistance. Prior work has shown that genetic variation in MAVS is associated with differences in antiviral signaling and some disease phenotypes in humans. For example, loss-of-function variants, such as C79F (rs11905552), are associated with reduce type I IFN and proinflammatory responses in some HIV patients. [40,41]. Additional studies have linked variants in the MAVS coding sequence to their ability to respond to poly-I:C in cultured cells—a surrogate for viral dsRNA [42], and in patient’s symptoms from Rift Valley Fever Virus infection [43]. Taken together, these findings suggest that MAVS polymorphisms may contribute to inter-individual differences in the ability to establish a PI and sensitivity to immune effectors, including C’, by modulating the strength and quality of MAVS-dependent signaling.

## Figures and Tables

**Table 1:** Nucleotide primers used for q-PCR.

Name	F’ Primer	R’ Primer
GAPDH	5′-TTAAAAGCAGCCCTGGTGAC-3′	5′-CTCTGCTCCTGTTCGAC-3′
F	5′-ACGTGTTATGGTGACTGGCA-3′	5′-GAACAGCACGAATCGAGTGA-3′
HN	5′-TGACCAACCCTTCGTCTACC-3′	5′-CTTGACCGCTTGATCCAAAT-3′
NP	5′-TGACCAGTCACCAGAAGCTG-3′	5′-CGGAATCAACGAAAGGTGTT-3′
IFNβ	5′-CAGCTCTTTCCATGAGCTACAA-3′	5′-CAGTATTCAAGCCTCCCATTCA-3′
IFIT1	5′-ACAGCAACCATGAGTACAAATGG-3′	5′-CATCGTCATCAATGGATAACTCCC-3′
OAS2	5′-AGAAGCTGGGTTGGTTTATC-3′	5′-GACGTCACAGATGGTGTTC-3′

## Data Availability

The original data are available upon request. Further inquiries can be directed to the corresponding author.
